# Towards a novel bioelectrocatalytic platform based on “wiring” of pyrroloquinoline quinone-dependent glucose dehydrogenase with an electrospun conductive polymeric fiber architecture

**DOI:** 10.1038/srep19858

**Published:** 2016-01-29

**Authors:** Johannes Gladisch, David Sarauli, Daniel Schäfer, Birgit Dietzel, Burkhard Schulz, Fred Lisdat

**Affiliations:** 1 Biosystems Technology, Institute for Applied Life Sciences, Technical University of Applied Sciences Wildau, Hochschulring 1, D-15745, Wildau, Germany; 2 Institute for Thin Film and Microsensor Technologies, Kantstraße 55, D-14513 Teltow, Germany; 3 Department of Chemistry and Centre for NanoScience (CeNS), University of Munich (LMU), Butenandtstraße 5-13 (E), D-81377, Munich, Germany

## Abstract

Electrospinning is known as a fabrication technique for electrode architectures that serve as immobilization matrices for biomolecules. The current work demonstrates a novel approach to construct a conductive polymeric platform, capable not only of immobilization, but also of electrical connection of the biomolecule with the electrode. It is produced upon electrospinning from mixtures of three different highly conductive sulfonated polyanilines and polyacrylonitrile on ITO electrodes. The resulting fiber mats are with a well-retained conductivity. After coupling the enzyme pyrroloquinoline quinone-dependent glucose dehydrogenase (PQQ-GDH) to polymeric structures and addition of the substrate glucose an efficient bioelectrocatalysis is demonstrated. Depending on the choice of the sulfonated polyanilline mediatorless bioelectrocatalysis starts at low potentials; no large overpotential is needed to drive the reaction. Thus, the electrospun conductive immobilization matrix acts here as a transducing element, representing a promising strategy to use 3D polymeric scaffolds as wiring agents for active enzymes. In addition, the mild and well reproducible fabrication process and the active role of the polymer film in withdrawing electrons from the reduced PQQ-GDH lead to a system with high stability. This could provide access to a larger group of enzymes for bioelectrochemical applications including biosensors and biofuel cells.

The organic nature of conductive electroactive polymers such as polyaniline, polypyrrole or polythiophene provides a good basis for the construction of electrodes that enable efficient communication with biosystems^1^,^2^,^3^. As a consequence of this fact they became significantly important as materials for the development of biosensors, since they can serve as immobilization matrices or represent transducing layers, the conductivity of which can be modified by a binding event^4^,^5^,^6^. Moreover, they can represent labels for binding partners or act as molecular wiring agents connecting biocatalysts with electrodes^4^,^5^,^6^.

Particularly polyanilines have been devoted attention since they show some advantages over other conducting polymers. They are of low-cost and easy to synthesize, being environmentally and thermally stable^7^,^8^,^9^. Furthermore, they can form dense, ordered or differently structured films, which possess a defined charge and are capable of binding or entrapment of biomolecules^10^,^11^,^12^,^13^,^14^. Customizing polyanilines’ properties by introducing of functional groups, e.g. upon sulfonation, one could not only increase their conductivity and solubility, but also improve their redox activity over a wide pH range, compared to unsubstituted polyanilines^7^. While sulfonated polyanilines are soluble in majority of solvents, unsubstituted ones often require additional substances for a proper processability^7^. In addition, the negative charge of the sulfonic acid group may be advantageous for the interaction with proteins or enzymes^13^. Several studies suggest that sulfonated polyanilines provide a suitable environment for the immobilization of redox proteins such as cytochrome c^15^,^16^,^17^ and hemoglobin^18^ as well as various redox enzymes, as e.g. sulfite oxidase^19^, peroxidase^20^,^21^, urease^22^, glucose oxidase^23^, or tyrosinase^24^. For some enzymes they also allow a wiring of the enzyme with the electrode^25^,^26^,^27^.

As electrochemical biosensor properties are dependent on the overall sensing surface, an enlarged electrode area is desirable. The enlargement can be achieved, for instance, by roughening the electrode or by creating three-dimensional structures. For this purpose different techniques are applicable, including chemical vapor deposition, electro-polymerization or immobilization of nanoparticles. A rather new technique with this respect is electrospinning, by means of which vastly enlarged surfaces can be achieved by the fabrication of architectures consisting of polymer fibers with diameters ranging from a few hundred nanometers to micrometers^28^,^29^. Many applications for electrospun fiber mats from medical diagnostics, and tissue engineering to materials for super capacitors or air filters have already been shown^30^,^31^,^32^,^33^. Also examples for biosensor applications of electrospun fiber mats have been reported^34^,^35^,^36^,^37^. However until now the polymeric fiber networks are mainly used as an interface to accommodate a large amount of molecules. Therefore, it would be desirable to develop the application of the technique further in order to combine biomolecule fixation with the electrochemical connection to the electrode.

Common polymers applied in electrospinning are polyacrylonitrile (PAN), polyethylene oxide (PEO) or polyvinyl alcohol. Modification of the fiber properties can not only be achieved by spinning mixtures of different polymers^30^,^38^,^39^, but also by addition of small particle-type of materials such as e.g carbon nanotubes to the polymer solution to be spun. The latter approach is also relevant when high conductivity is desired^38^,^40^,^41^. In this study we demonstrate the fabrication of conducting 3D polymer fiber networks by electrospinning of 3 different chemically synthesized sulfonated polyanilines together with non-conducting PAN. These electrodes have been initially characterized by means of microscopy and electrochemistry of a small-size, inorganic redox couple. Subsequently, the enzyme pyrroloquinoline quinone-dependent glucose dehydrogenase (PQQ-GDH) has been covalently bound to the polymer electrodes and the shuttle-free bioelectrocatalytic behavior of the system has been investigated.

## Results and Discussion

**Figure 1:**
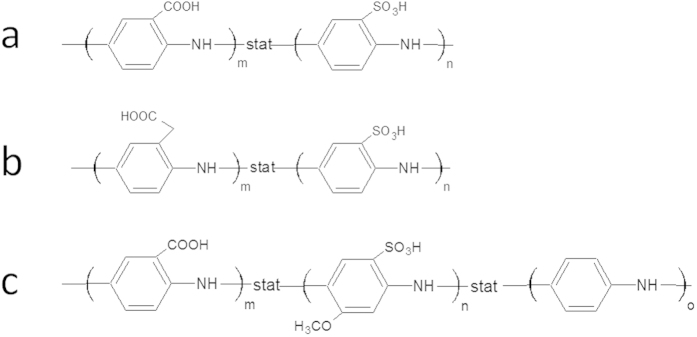
Chemical structure of different sulfonated polyanilines used : (**a**) PABSA, (**b**) PAPASA, (**c**) PANABMSA.

Sulfonated polyanilines have been chosen for the electrospinning process because of their conductive properties and since the polymers directly interact with the enzyme PQQ-GDH allowing direct electron transfer between the substrate reduced enzyme and the polymer^42^. Three different sulfonated polyanilines (PABSA [poly(3-aminobenzoic acid-co-3-aminobenzenesulfonic acid)], PAPASA [poly(3-aminobenzeneacetic acid-co-3-aminobenzenesulfonic acid)], PANABMSA [poly(3-aminobenzoic acid-co-2-methoxyaniline-5-sulfonic acid-co-aniline)], Fig. 1) have been used. Apart from sulfonic acid groups, the polymers exhibit carboxylic acid groups, which allow a covalent fixation of the enzyme PQQ- GDH. Differences among the polymers occur due to the presence of different substitutions on the aniline ring (namely methoxy-, ethylcarboxy- and carboxy- groups) or the presence of unsubstituted aniline during copolymerisation.

### Characterization of the Copolymers

**Figure 2:**
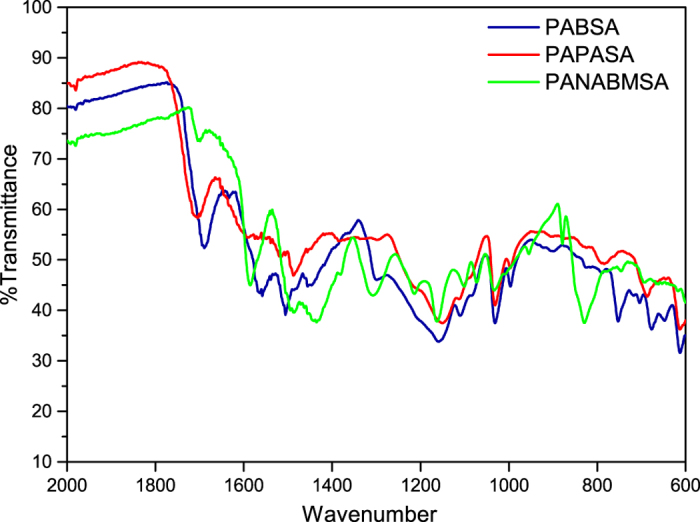
IR-spectra of PABSA, PAPASA and PANAMBSA polymers.

The synthesized polymers have been characterized by means of gel permeation chromatography (GPC), thermogravimetral analysis (TGA), and IR spectroscopy. The results are presented in Table 1, and the IR spectra are shown in Fig. 2. There, the bands in the range of 3000–3500 cm^−1^ correspond to stretching vibration N-H bonds for all polymers (not shown). The most prominent absorption band of the carbonyl group of carboxylic acids is found between 1704 cm^−1^ and 1689 cm^−1^. Further bands at 1558–1585 cm^−1^ and 1488–1506 cm^−1^ correspond to the ring stretching vibration of the quinoid and benzenoid rings. All polymers show a more intense benzenoid band compared to the quinoid band. The C-N stretching mode gives rise to a band near 1448–1451 cm^−1^. The band at 1293–1300 cm^−1^ is related to the C-N stretching vibration of aromatic amines associated with para-linked aniline units^43^. The bands observed at 1143–1158 cm^−1^ are primarily due to the aromatic C-H in-plane deformation vibration. The intense band near 1031 cm^−1^ is attributed as the symmetric stretching vibration of S=O. The sharp bands at 612–615 cm^−1^ and 687–693 cm^−1^ are associated with the C-S and S=O stretching vibrations. Bands of the C-H out-of-plane bending vibrations at 869–878 cm^−1^, 825–830 cm^−1^ and 796–798 cm^−1^ corresponding to 1,2,4 and 1,4-substituted benzene rings indicating that the monomers in the polymers are connected in head-to-tail fashion^44^. The presence of tetra1,2,4,5 substituted benzene rings has been identified by the appearance of characteristic bands at 851 cm^−1^

### Fabrication and characterization of the 3D electrodes

Attempting to electrospin the polyanilines, we have found that the used polyanilines cannot be processed in electrospinning alone, since electrospraying occurs, probably due to relatively low viscosity and molecular weight of these polymers. Thus, mixtures of sulfonated polyanilines and polyacrylonitrile (PAN) have been used for the electrode fabrication. In order to get a 3D network allowing both electrochemical and optical investigations, the electrospun fiber networks have beenprepared on planar indium tin oxide (ITO) electrodes. After preparation they have been initially characterized by light microscopy in order to check the general quality of polymer networks and to evaluate their thickness (see Supporting Information, Figure S1). Subsequently, the polymer electrodes are characterized by means of scanning electron microscopy.

**Figure 3:**
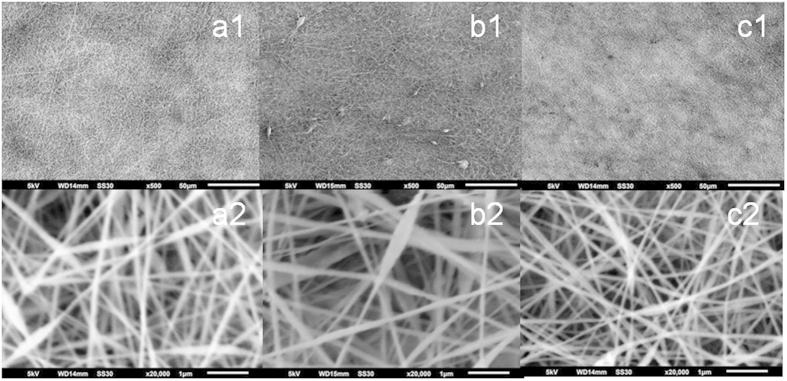
SEM images of the fiber morphology in the electrospun mats of (**a**) ITO-PAN/PAPASA, (**b**) ITO-PAN/PANABMSA, (**c**) ITO-PAN/PABSA, whereas 1 and 2 correspond to different magnifications used (with lines represent 50 μm and 1 μm, respectively).

Figure 3 presents light microscopy and SEM images of PAN/PAPASA, PAN/PANAMBSA and PAN/PABSA composites fabricated from a mixed polymer solution in DMSO. Prior to the spinning process all polymer-containing solutions have been ultrasonicated. Once dispersed, they remain stable in DMSO for at least 3 weeks; neither sedimentation nor aggregation of polymers have been observed. A flow rate of 2 μl/min is chosen for the electrospinning procedure at almost no dripping. Depending on the chosen polyaniline, the polymer fiber mats show a brownish (Figure S1: a1 - PAN/PAPASA and c1 - PAN/PABSA) or green-bluish (Figure S1: b1 - PAN/PANABMSA) color, which is in agreement with the different redox states (and thus color) of the polyanilines after synthesis. The polymer fiber networks have found to be uniform in their appearance. This is valid for the mats from different polymeric blends and also among multiple electrodes modified with the same polymer mix. Only in some few cases fiber agglomeration could be observed - particularly for fibers made of PAN and PANABMSA, most probably because of the higher viscosity of PANAMBSA compared to other polymers.

**Figure 4:**
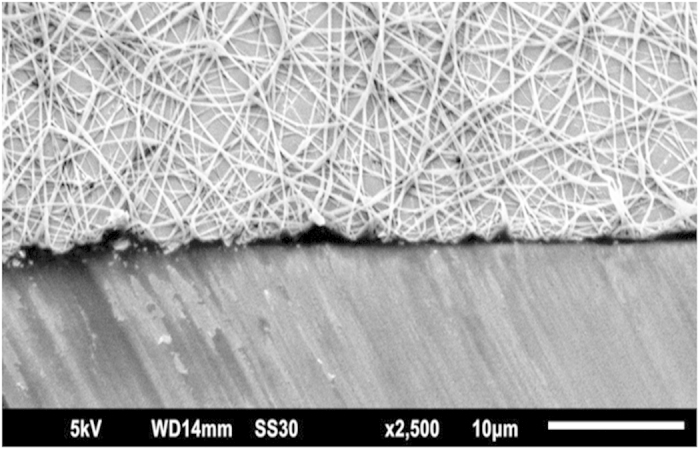
SEM image of an ITO-PAN/PABSA mat after 15 minutes electrospinning.

Figure 4 shows a cut edge of a PAN/PABSA polymer fleece prepared in a 15 min electrospinning process indicating a proper mat thickness in the range of few micrometers. The averaged diameters of the fibers are shown in Table 2. In case of PAN/PANABMSA the mean diameters as well as the standard deviations are slightly larger. Moreover, even when the fibers of all polymer mixtures show a slightly beaded structure, the averaged bead diameters were significantly larger in case of PAN/PANABMSA. As the bead formation is influenced by many parameters, especially concentration and voltage, the relatively high voltage of 25 kV might be the reason for bead formation^45^,^46^. As for the interaction with water, all mats showed a sponge-like behavior. For instance, when contact angle measurements are conducted, the polymer fiber mats give small angles mostly about 30 °.

### Electrochemical characterization of the fiber mats

**Figure 5:**
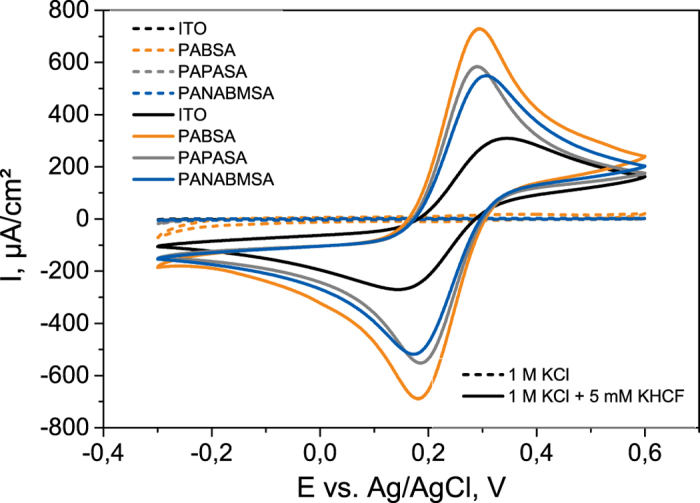
Cyclic voltammograms of differently modified electrodes obtained at a scan rate of 100 mV/s. Measurements were conducted with 1 M KCl and 1 M KCl + 5 mM K_4_[Fe(CN)_6_]/ K_3_[Fe(CN)_6_] respectively. The polymer fibers were deposited on ITO surfaces by means of electrospinning for 15 minutes.

In order to evaluate whether the electrospun network can operate as electrode interface cyclic voltammetric investigations in the presence of the [Fe(CN)_6_]^4−/3−^ redox couple have been performed. In comparison to the bare ITO electrode, which served as the basic material, a clearly enhanced conversion of the redox ions is found for all polymer fiber electrodes. This indicates on the one hand sufficient conductivity and on the other hand a surface which is suited to electrocatalytic conversions. Comparing different polymer electrodes (Fig. 5 and Table 3), PAN/PABSA gives the highest current values. PAN/PAPASA and PAN/PABSA show smaller peak separations than PAN/PANABMSA (all electrospun for 15 min). Moreover, these peak separations are all lower than for a bare ITO electrode indicating a faster kinetics. In case of the electrodes with different spinning durations (and thus different thicknesses - studied for PAN/PABSA), both 5 and 30 min spun electrodes show a higher peak separation compared to 15 min. Concerning the E_f_ value, no difference between all the different preparations have been determined. Furthermore, heterogeneous electron transfer rate constants (k_s_) are calculated according to the Nicholson and Shain approach (Table 3)^47^. The lowest k_s_ value has been determined in case of the bare ITO electrodes, demonstrating that the 3D polymer mats are beneficial in terms of electron transfer rate. Comparing the values for different polymers electrospun for 15 minutes, PAN/PABSA shows the lowest value and smallest error, whilst both k_s_ values for PAN/PAPASA and PAN/PANABMSA are slightly higher but with higher errors. Taking the peak current maxima and the kinetics in account one can conclude that all three polymer networks provide a suitable interface for electrochemical conversions.

### Bioelectrocatalysis of PQQ-GDH at the polymer fiber network

To evaluate the effect of the electrospun networks as efficient conductive supports for enzyme immobilization, the resulting electrodes have been modified by the enzyme PQQ-GDH. The enzyme has been chosen since different applications of PQQ-GDH with surface structures have been demonstrated as e.g. in sensing, substance release and bioenergetics^48^,^49^,^50^. Here, the enzyme has been covalently bound to the carboxylic acid groups on the aniline rings (Fig. 1). The attractive feature of the sulfonated polyaniline is the conductivity at neutral pH. Moreover, we have already reported on an efficient reaction of PQQ-GDH with these kind of polymers in solution but also on the communication with electrodes modified by similar sulfonated polyaniline copolymers^26^. The approach of covalent fixation of the enzyme in this study has been mainly chosen to ensure a good stability of the enzyme electrodes.

**Figure 6:**
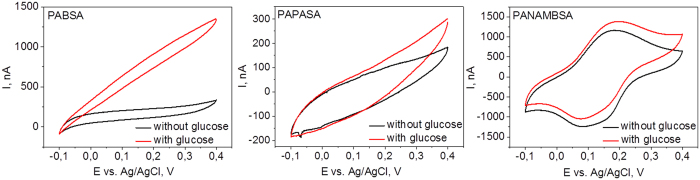
CVs of ITO-PAN/PABSA, ITO-PAN/PAPASA and ITO-PAN/PANAMBSA with covalently fixed PQQ-GDH without (black lines) and after addition of 5 mM of glucose (red lines). Experimental conditions: [glucose] = 5 mM in 20 mM MES buffer at pH 6, t = 25 °C, scan rate 5 mV/s.

Figure 6 summarizes cyclic voltammograms of electrospun PAN/PABSA, PAN/PAPASA and PAN/PANAMBSA electrodes in the absence and in the presence of the substrate glucose. In the absence of glucose no redox conversion of polymers PABSA and PAPASA can be observed, whereas a clear redox couple with a formal potential about *E* = +0.14 V vs. Ag/AgCl can be measured for PANAMBSA, being in good agreement with already reported values for sulfonated polyanilines^26^,^51^,^52^. The different voltammetric behavior might be attributed to different initial redox states of copolymers after synthesis (PABSA and PAPASA are in fully oxidized pernigraniline state, whereas PANAMBSA is in a half-oxidized emeraldine salt state) and has also been found in previous studies^8^,^26^,^53^,^54^. Nevertheless, in all three cases significant bioelectrocatalytic currents appear upon addition of glucose. When a bare ITO electrode is incubated in a PQQ-GDH solution and subsequently studied in glucose containing buffer no bioelectrocatalysis could be found, demonstrating the role of the electrospun polymer matrix in the enzymatic reaction.

However, the bioelectrocatalytic signals differ both by their starting potentials and the current intensities (Table 4). If a bioelectrocatalytic current after addition of the substrate is observed at potentials corresponding to the redox conversion of the polymer, mediation can be concluded. In case of ITO-PAN/PANAMBSA electrodes this mechanism might be suggested, since the bioelectrocatalytic current appears at *E* = +0.1 V vs. Ag/AgCl indicating that the oxidation process of the polymer may trigger the bioelectrocatalysis. However, for ITO-PAN/PABSA and ITO-PAN/PAPASA bioelectrocatalysis is observed at lower potentials (Table 4), leading to the conclusion that a direct electron transfer between the suitably oriented enzyme and polymer-modified electrode surface is valid. This is in agreement with already reported starting potentials of PQQ-GDH catalysis at electrodes modified with polymers^13^,^26^,^55^,^56^ or other surfaces.^48^,^49^,^58^,^59^

From the data in Table 4 it is obvious, that depending on used polymers, significantly different intensities of catalytic currents can be observed. ITO-PAN/PAPASA electrodes exhibit lowest bioelectrocatalytic activity. In contrast the intensity of the. glucose-induced catalytic current in the case of PAN/PABSA is found to be 10fold higher than that of ITO-PAN/PAPASA, making ITO-PAN/PABSA electrodes to the most promising candidate for efficient enzyme connection to electrodes and furthermore for potential biosensorial utilization.

In order to optimize the performance of PQQ-GDH-modified 3D polymer mats the electrospinning time has been varied from 15 via 30 to 60 minutes (prior to enzyme fixation). The bioelectrochemical results for these electrodes are summarized in Table 5. It can be clearly concluded that the increase in electrospinning time leads to a slight decrease in catalytic activity of the enzyme electrodes, demonstrating that thinner polymer mats are more suitable for the preparation of electroactive electrodes. Although thicker polymer layers can provide more interaction sites for the immobilization of the enzyme the transport of glucose may limit the overall process. The slight decrease in catalytic current may also be attributed to the large distances for electron transport within the rather thick polymeric mats. Here one has to take into account that a large portion of the fibers is composed of the non-conducting PAN polymer. Thus, the efficient bioelectrocatalysis obtained with these fibers from polymeric blends can be considered a real success.

**Figure 7:**
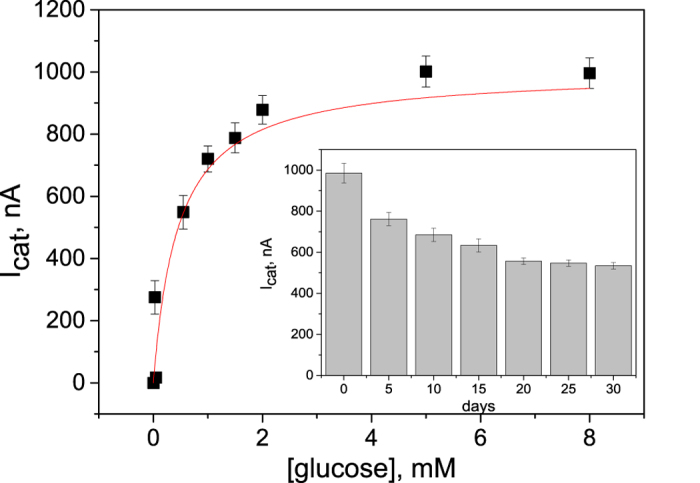
Catalytic current response of the ITO-PAN/PABSA/PQQ-GDH electrode (15 minutes electrospinning time) as a function of the glucose concentration. Bioelectrocatalytic signals were registered from the respective cyclic voltammograms at *E* = +0.35 V vs Ag/AgCl. Inset: Stability of the electrode response towards 5 mM glucose. Experimental conditions: 20 mM MES buffer pH 6, CV at 5 mV s^−1^, n = 3.

Figure 7 demonstrates a calibration curve obtained for ITO-PAN/PABSA electrodes (n = 3) by evaluating the oxidation current as a function of the glucose concentration at *E* = +0.35 V vs Ag/AgCl. For glucose concentrations from 0.0025 to 1 mM a linear response is found. From 2 mM to 8 mM a saturation behavior is observed. An apparent K_m_ value of 0.45 mM glucose can be calculated based on the Michaelis-Menten kinetics. The concentration dependent behavior shows that the system allows a well-defined detection of glucose, but it also demonstrates that that at higher glucose concentration the enzyme electrode cannot follow anymore and the electron withdrawal from the reduced enzyme becomes rate limiting.

Considering the blood glucose level in the reference range between 3.6– 5.6 mM one can conclude that the present biosensing system has a potential to be implemented to monitoring the glucose level in human blood. It has also to be explicitly noted here that glucose detection can be performed at low potentials because bioelectrocatalysis already starts at −0.1 V vs Ag/AgCl. This is advantageous in complex media, in which other redox-active substances are present. However, limited glucose selectivity of PQQ-GDH is still an issue to be overcome with this respect^60^.

Since long-term stability is an important parameter for the evaluation of the performance of any sensing system, we have traced the stability of our ITO-PAN/PABSA-PQQ-GDH electrodes by testing their activity in glucose solution, after the electrodes were kept at 4 °C in 20 mM MES, pH 6 when not in use. Inset in Fig. 7 shows that the catalytic current density response is maintained over 65% of its initial value after 30 days. The signal decline might be caused by loss in PQQ-GDH activity rather than by leakage of the enzyme from the electrode. Although such kind of bioelectrocatalytic platform is mostly desirable for a single-use application, the improved long-term stability makes this type of architecture promising to be considered for the construction of glucose detection systems.

## Conclusion

In this report the successful fabrication of conducting 3D polymer fiber networks by electrospinning of 3 different, chemically synthesized sulfonated polyanilines together with non-conducting polyacrylonitrile has been demonstrated. Microscopic characterizations of PAN/PABSA, PAN/PAPASA and PAN/PANAMBSA mats have shown that the fibers are uniformly distributed within the network on the ITO surface. The electrochemical characterization of these electrodes in the presence of hexacyanoferrate (II/III) reveals that the polymeric network allows electrochemical conversions to occur. Compared to a bare ITO electrode higher Faradayic currents and faster electron transfer are found.

Moreover, the polymer fibers provide a surface for fixation of active enzyme as exemplified with PQQ-GDH. Significant bioelectrocatalytic currents occur upon addition of glucose to 3D polymer mats with the covalently-bound enzyme. Depending on the choice of the sulfonated polyanilline mediatorless bioelectrocatalysis can be obtained, starting at quite low potentials; i.e., no large overpotential is needed to drive the reaction. A well-defined concentration dependent response is observed for PAN/PABSA. In addition, the mild and well reproducible fabrication process and the active role of the polymer film in withdrawing electrons from the reduced PQQ-GDH lead to a system with high stability. Thus, such electrospun networks developed by making use of a sulfonated polyaniline and its interaction with PQQ-GDH can be considered as a promising candidate for biosensorial purposes.

## Methods

### Materials

If not otherwise stated, all reagents and solvents have been used without further pretreatment. Dimethyl sulfoxide (DMSO), CaCl_2_, 2-(N-morpholino)ethanesulfonic acid (MES), polyacrylonitrile and potassium ferricyanide were provided by Sigma Aldrich (Sigma, Aldrich, Sigma Aldrich, Fluka). Acetone, ethanol and potassium ferrocyanide were bought from Roth (CARL ROTH GmbH und CO. KG, Germany). 3-Aminobenzoic acid (3-AB) (Sigma-Aldrich Chemicals Co., Germany) and 3-aminobenzenesulfonic acid (3-ABS) (Sigma-Aldrich Chemicals Co. ,Fluka, Germany) were recrystallized from water (3-AB: m.p. 176–177 °C; 3-ABS: m.p. 288 °C). 3-Aminobenzeneacetic acid (3-ABA), 3-Amino-4-methoxybenzenesulfonic acid (MAS) (Sigma-Aldrich Chemicals Co., Germany), ammonium persulfate (APS), HCl, DMF, and sodium hydroxide (Sigma-Aldrich Chemicals Co., Fluka, Germany) were used as purchased. Ultra clear water was produced with a SG water ultra pure water unit.

## Copolymer Synthesis

### PAPASA

was prepared by chemical oxidative polymerization according the following procedure: 1 g (0,006 mol) 3-aminobenzeneacetic acid and 1,14 g (0,006 mol) 3-aminobenzenesulfonic acid were dissolved in 27 ml 0,5 M NaOH. 2,97 g (0,013 mol) ammonium persulfate dissolved in 9 ml water added slowly under continuous stirring for more than 30 min at room temperature. The reaction mixture was stirred for further 24 hrs at room-temperature to complete the reaction. The precipitated product was filtered, washed with aqueous 1.2 M HCl and water and dried under vacuum at 80 °C for 24 h.

### PANABMSA

was synthesized as follows: A 1:1 mixture of 80 ml 0,5 M NaOH containing 8,1 g (0,04 mol) 3-amino-4-methoxybenzene sulfonic acid, 10 ml 0,5 M NaOH containing 0,55 g (0,004 mol) 3-aminobenzoic acid and 3,72 g (0,04 mol) Aniline was stirred at room temperature. 12,3 g (0,054 mol) ammonium persulfate dissolved in 30 ml water added slowly for more than 30 min. The reaction mixture was stirred for further 24 hrs at room-temperature to complete the reaction. The precipitated product was filtered, washed with aqueous 1.2 M HCl and water and dried under vacuum at 80 °C for 24 h.

### PABSA

was synthesized as previously reported^26^.

### Polymer Characterization

The molecular weights were determined by gel permeation chromatography (GPC) using a Waters apparatus provided with refraction and UV detectors and PL Mixed Gel C Column. Measurements were carried out in DMF using polystyrene standards. IR spectra were recorded with Bio-Rad FTX 3000MX spectrometer. ^1^H-NMR measurements were carried out with a Bruker Instrument. Thermogravimetral analysis (TGA) was performed by Perkin Elmer TG 7 apparatus.

### Preparation of the polymer solution

For the preparation of the polymer solution 50 mg/ml polyacrylonitrile and 10 mg/ml of a polyaniline derivative were dissolved in DMSO and ultrasonicated for 11 hours.

### Electrospinning

Prior to the preparation of the polymer fibers, the rectangular indium tin oxide (ITO) coated glass slides with surface resistivity 15–25 Ω sq^−1^ (obtained from Sigma-Aldrich, Taufkirchen, Germany) were cleaned processing DMSO-water-acetone-water-ethanol solvent steps (15 min ultrasonication for each step). The cleaned electrodes were then placed into the cabin of the electrospinning apparatus EC-CLI (IME Technologies, The Netherlands), and the polymer solution was electrospun onto their surfaces from a distance of 35 cm at a voltage of 25 kV with 2 μl/min spinning rate. The temperature was controlled to be 25 °C and the humidity of 40%. For the spinning process a 27G1” needle (B Braun Melsungen, Germany) was used. The electrospinning procedure was conducted for different times from 5 to 60 min.

### Characterization of polymer fiber mats

First the coated electrodes were investigated by means of light microscopy (Olympus DSX500, Olympus Deutschland GmbH, Germany) to confirm the overall structure and uniformity. Additionally the polymer fiber mat coated electrodes where investigated with an electron microscope (JEOL JSM-6510 SEM, JEOL Germany GmbH, Germany) at a voltage of 5 kV. Images where taken at different areas of the electrodes and the diameters of the fibers were measured. Contact angle measurements were conducted with a Dataphysics OCA15EC machine (DataPhysics GmbH, Germany).

### Preparation of the enzyme solution

Soluble glucose dehydrogenase (apoGDH) was gained as a kind gift from Roche. The enzyme was dissolved in 5 mM MES buffer in the presence of 1 mM CaCl_2_, and the pH was adjusted to 5. apoGDH was reconstituted with PQQ in a ratio of 1:1 according to Olsthoorn and Duine^57^. For this purpose, sGDH and PQQ were incubated together for 1 h at room temperature in the dark. Aliquots were stored at −20 °C. Prior to each measurement, the specific activity of the reconstituted enzyme was determined to be 2200 ± 30 U mg^−1^ by using 2,6-dichlorophenolindophenol as an electron acceptor.

### Enzyme binding

The ITO electrodes on which the electrospun polymer network has been fabricated, have been used to covalently bind PQQ-GDH. In order to fix the enzyme covalently, firstly the polymer-modified electrodes were placed in an EDC/NHS-solution (100 mM/25 mM) in 5 mM MES + 1 mM CaCl_2_, pH 6.5 for 15 minutes and after that washed 3 times with the same buffer before enzyme incubation. For the incubation purpose polymer electrodes have been placed in enzyme solution for 1 h, afterwards washed with buffer prior measurements.

### Electrochemistry

The electrodes were characterized electrochemically by means of cyclic voltammetry using a CH Instruments 1230b in a custom made 1 ml measurement cell. A polymer-modified ITO electrodes with the effective electrode area of 0.32 cm^2^ (Figure S2, Supporting Information) were used as working electrodes, together with an Ag/AgCl/1 M KCl reference (Biometra, Germany) and a Pt-wire counter electrodes. Electrochemical characterizations were performed in the presence of K_4_[Fe(CN)_6_]/ K_3_[Fe(CN)_6_] (5 mM) in 1 M KCl.

Bioelectrochemical measurements were performed at room temperature in the same cell using a 20 mM MES buffer pH 6 as electrolyte. The catalytic response was studied with addition of different glucose concentrations to the solution. Cyclic voltammetric experiments were carried out with a μAutolab Type II device (Metrohm, The Netherlands). The scan rate was set to 5 mV s^−1^. The potential range was chosen as between −0.4 and +0.4 V vs. Ag/AgCl. Data analysis was performed using GPES software (General Purpose for Electrochemical System, Eco Chemie, The Netherlands).

## Additional Information

**How to cite this article**: Gladisch, J. *et al.* Towards a novel bioelectrocatalytic platform based on “wiring’’ of pyrroloquinoline quinone-dependent glucose dehydrogenase with an electrospun conductive polymeric fiber architecture. *Sci. Rep.* **6**, 19858; doi: 10.1038/srep19858 (2016).

## Supplementary Material

Supplementary Information

## Figures and Tables

**Table 1:** Composition, sulfonation grade, yields, molecular weights and the polydispersity index (PDI) values of the copolymers used in this study.

Polymers	C [%]	H [%]	N [%]	S [%]	S/N	Yield [%]	M_n_^[g/mol]^	M_w_^[g/mol]^	PDI
PAPASA	49,88	3,86	7,22	3,66	0,22	30	7500	11000	2,06
PANABMSA	51,19	3,78	9,12	2,58	0,12	42	n. d.	n. d.	n. d.
PABSA	48,79	3,88	7,72	4,24	0,24	19	8400	11200	1,30

**Table 2:** Averaged diameter and bead diameter of different fiber mats as a result of the characterization by electron microscopy (n = 3).

	ITO-PAN/PAPASA	ITO-PAN/PANABMSA	ITO-PAN/PABSA
diameter [μm]	0,13 ± 0,02	0,15 ± 0,04	0,12 ± 0,02
bead diameter [μm]	0,31 ± 0,08	0,51 ± 0,15	0,30 ± 0,05

**Table 3:** Results of the electrochemical investigations (peak separations, formal potentials and heterogeneous electron transfer rates) of the fiber mats on ITO surfaces performed at 100 mV/s in 1 M KCl + 5 mM K_4_[Fe(CN)_6_]/ K_3_[Fe(CN)_6_] prepared by electrospinning for 15 min.

electrodes	ΔE_p_ [V]	E_f_ [V]	k_s_ × 10^3^, [cm/s]
PAN/PAPASA	0,104 ± 0,007	0,239 ± 0,002	9,5 ± 3,6
PAN/PANABMSA	0,139 ± 0,043	0,244 ± 0,004	8,9 ± 3,6
PAN/PABSA	0,113 ± 0,006	0,239 ± 0,002	6,4 ± 1,1
bare ITO	0,186 ± 0,052	0,244 ± 0,002	5,6 ± 2,5

**Table 4:** Start potentials of bioelectrocatalysis and current intensities after glucose addition (5 mM) at 0,35 V vs Ag/AgCl of ITO-PAN/PABSA, ITO-PAN/PAPASA and ITO-PAN/PANAMBSA electrodes prepared by 15 minutes electrospinning.

electrodes	starting potential, *E* vs. Ag/AgCl, V	current intensity, nA
ITO-PAN/PABSA	−0.1	980 ± 50
ITO-PAN/PAPASA	−0.05	98 ± 30
ITO-PAN/PANAMBSA	+0.1	360 ± 40

**Table 5:** Electrospinning time and its influence on catalytic current intensities of ITO-PAN/PABSA, ITO-PAN/PAPASA and ITO-PAN/PANAMBSA electrodes in the presence of 5 mM glucose determined at +0,35 V vs Ag/AgCl (n = 3).

electrodes	electrospinning time, min	current intensity nA
ITO-PAN/PABSA	30	807 ± 30
	60	630 ± 40
ITO-PAN/PAPASA	30	74 ± 20
	60	51 ± 10
ITO-PAN/PANAMBSA	30	293 ± 50
	60	226 ± 30
