# Supplementary material for: Towards a novel bioelectrocatalytic platform based on “wiring” of pyrroloquinoline quinone-dependent glucose dehydrogenase with an electrospun conductive polymeric fiber architecture
Source: Sci Rep. 2016 Jan 29;6:19858. doi: 10.1038/srep19858 (PMC4731776; doi:10.1038/srep19858)

**SUPPORTING INFORMATION**

Towards a novel bioelectrocatalytic platform based on "wiring" of pyrroloquinoline quinone-dependent glucose dehydrogenase with an electrospun conductive polymeric fiber architecture

Johannes Gladisch^1^‡, David Sarauli^1,3^‡^*^, Daniel Schäfer^1^, Birgit Dietzel^2^, Burkhard Schulz^2^, Fred Lisdat^1^

^1^ Biosystems Technology, Institute for Applied Life Sciences, Technical University of Applied Sciences Wildau, Hochschulring 1, D-15745, Wildau, Germany; ^2^ Institute for Thin Film and Microsensor Technologies, Kantstraße 55, D-14513 Teltow, Germany; ^3^ Department of Chemistry and Centre for NanoScience (CeNS), University of Munich (LMU), Butenandtstraße 5-13 (E), D-81377, Munich, Germany

‡Both authors contributed equally to the presented study.

**Figure S1.** Light microscopy images for a) ITO-PAN/PAPASA, b) ITO-PAN/PANAMBSA and c) ITO-PAN/PABSA polymer fiber networks prepared by electrospinning on ITO electrodes. The yellow line represents 500 µm.


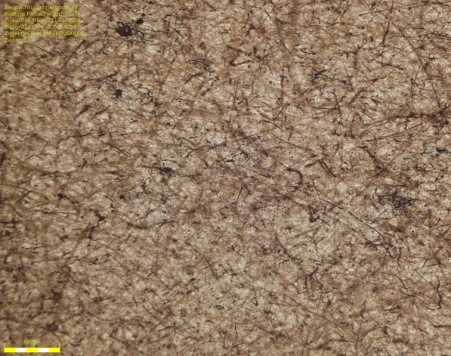

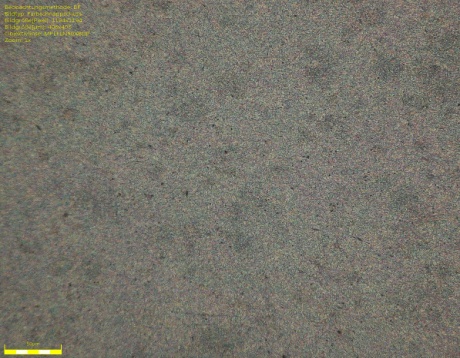

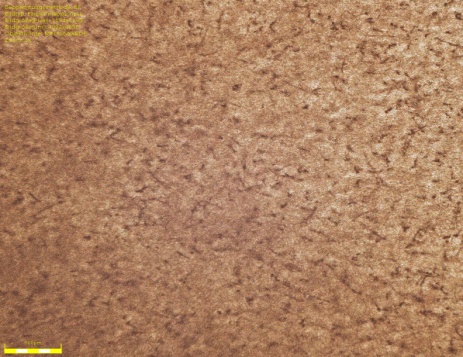


a

b

c

a2

b2

c2

**Figure S2**. Schematic view of the ITO electrodes covered with the different polymer fiber networks prepared by electrospinning. To ensure defined electrochemical areas the polymer was partly removed resulting in a dimension of the active electrode surface of 0,32cm^2^.


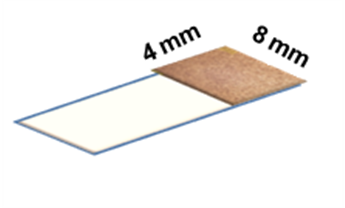

Supplement: Supplementary Information [file srep19858-s1.doc]
